# Synergistic Interface Engineering via Buffer Layer and UVO Treatment for High‐Performance PbS Quantum Dot Near‐Infrared Photodiodes

**DOI:** 10.1002/advs.76271

**Published:** 2026-06-25

**Authors:** Chuan Wei, Jun Han, Ning Feng, Li Yan, Hao Sun, Yuanhong Gao, Meili Xu, Guodan Wei, Hong Meng

**Affiliations:** ^1^ School of Advanced Materials Shenzhen Graduate School Peking University Shenzhen P. R. China; ^2^ School of Flexible Electronics (SoFE) Sun Yat‐sen University Shenzhen Guangdong P. R. China; ^3^ Institute of Materials Research Shenzhen Geim Graphene Center Shenzhen International Graduate School Tsinghua University Shenzhen P. R. China

**Keywords:** dual‐interface engineering, near‐infrared photodiodes, PbS quantum dots, Poly‐TPD buffer, UVO treatment

## Abstract

While PbS quantum dot (QD) photodetectors exhibit promising near‐infrared (NIR) response, their performance is limited by interfacial defects and damage caused by ligand‐exchange processes. This study introduces a dual‐interface engineering strategy: (I) A solution‐processable Poly‐TPD buffer layer is inserted between the PbS‐halide active layer and PbS‐EDT hole transport layer to mitigate EDT/acetonitrile‐induced interfacial damage and improve hole extraction; (II) A controlled UV‐ozone treatment is applied to the PbS‐EDT layer to enhance p‐doping density and reduce defect states. The optimized device achieves a dark current density as low as 74 nA cm^−^^2^ at ‐0.5 V, a responsivity of 0.42 A W^−1^ (at 1350 nm illumination), and a specific detectivity (D*) of 2.1 × 10^12^ Jones, placing it among the higher‐performing solution‐processed PbS CQD photodiodes operating in the 1.3–1.4 µm range. This work provides a scalable approach for developing high‐performance solution‐processed NIR photodetectors.

## Graphical abstract

A dual‐interface strategy combining a solvent‐orthogonal Poly‐TPD buffer with UV–ozone tuning of PbS‐EDT reconstructs interfacial energetics and defect chemistry in PbS CQD photodiodes. The optimized interface suppresses shunt‐ and trap‐related leakage, reduces noise, and enables high‐detectivity near‐infrared detection with imaging capability.

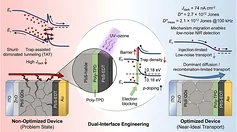

## 1 Introduction

Semiconductor quantum dots (QDs) are zero‑dimensional nanomaterials with size‑dependent optical and electrical properties, and they have become powerful building blocks for photodetection [1–5]. Among various QD semiconductors, PbS QDs are particularly attractive for near‑infrared (NIR) to short‑wave infrared (SWIR) applications [6] because their bandgap is widely tunable, their cost is low, and they are compatible with solution processing [7–9]. These advantages enable compact, scalable photodetectors for fiber‑optic communication, biomedical imaging, and defense‑relevant sensing [9–12]. Despite rapid progress, performance optimization remains challenging, especially in carrier transport, surface passivation, interfacial defect control, and dark current suppression [13, 14].

High‐performance PbS QD photodetectors commonly adopt a p‐i‐n architecture [15–19], in which the interfacial morphology between the electron‐transport layer (ETL) and the intrinsic active layer (i‐layer), as well as that between the i‐layer and the hole‐transport layer (HTL) [20], together with precise energy‐level alignment, strongly govern carrier transport and recombination dynamics [21, 22], and are therefore critical to overall device performance. During fabrication, 1,2‐ethanedithiol (EDT) is widely used as a ligand‐exchange reagent to passivate PbS QDs and form a PbS‐EDT HTL [23, 24]. However, thiol‐based EDT processing can be chemically reactive toward the PbS‐halide active layer; particularly under high ligand concentration, it severely degrades the surface morphology and increases the density of defect states [24, 25], thereby exacerbating carrier recombination and undermining extraction efficiency [26]. More importantly, EDT‐induced interfacial deterioration not only reduces device yield but also introduces uncontrolled performance fluctuations. In addition, the PbS‐EDT HTL itself often suffers from poor film quality, high defect density, and insufficient p‐type doping, which collectively manifest as elevated recombination centers and increased dark current. Zhao N [27], Kirmani A R [28], and Tang J [29] attempted to improve PbS QD detector performance via air exposure (boosting QD solar‐cell PCE by >2%), while Hwang G W [30] employed oxidant treatment (reducing the trap‐state density by 40‐fold). Nevertheless, these approaches generally lack precise controllability and are sensitive to ambient conditions (e.g., humidity), making it difficult to achieve stable, reproducible optimization. Consequently, the device's dark current often remains undesirably high.

To address these challenges, we develop a synergistic dual‐interface engineering strategy: (I) inserting an ultrathin, dense Poly‐TPD polymer buffer at the PbS‐halide/PbS‐EDT interface to protect the PbS‐halide absorber from EDT‐solvent erosion via solvent orthogonality, while enabling stepped band alignment for efficient hole extraction; and (II) applying a controlled UV‐ozone (UVO) treatment to the PbS‐EDT layer to reduce defect states and strengthen p‐type doping [31]. Poly‐TPD, a widely used hole‐transport material in organic light‐emitting diodes (OLEDs) [32, 33], offers well‐matched energetics with PbS‐halide (Valence Band Maximum (VBM) is 5.2 eV) and good compatibility with chlorobenzene processing, thereby preserving interfacial integrity [32, 34]; meanwhile, its low Conduction Band Minimum (CBM) (2.3 eV) relative to PbS provides effective electron blocking and suppresses electron injection [35]. UVO further enhances the p‐type character of thiol‐capped PbS QDs by inducing a controlled oxidation (Fermi‐level shift of 0.18 eV), thereby improving energy‐level alignment. With this cooperative interface design, the dark current density is suppressed by nearly two orders of magnitude to 74 nA cm^−^^2^, while the specific detectivity is enhanced to 2.1 × 10^1^ ^2^ Jones. These results place the device among the higher‐performing solution‐processed PbS CQD photodiodes operating in the 1.3–1.4 µm range and highlight the potential of this interface‐engineering strategy for high‐performance NIR photodetectors.

## 2 Results and Discussion

**FIGURE 1:**
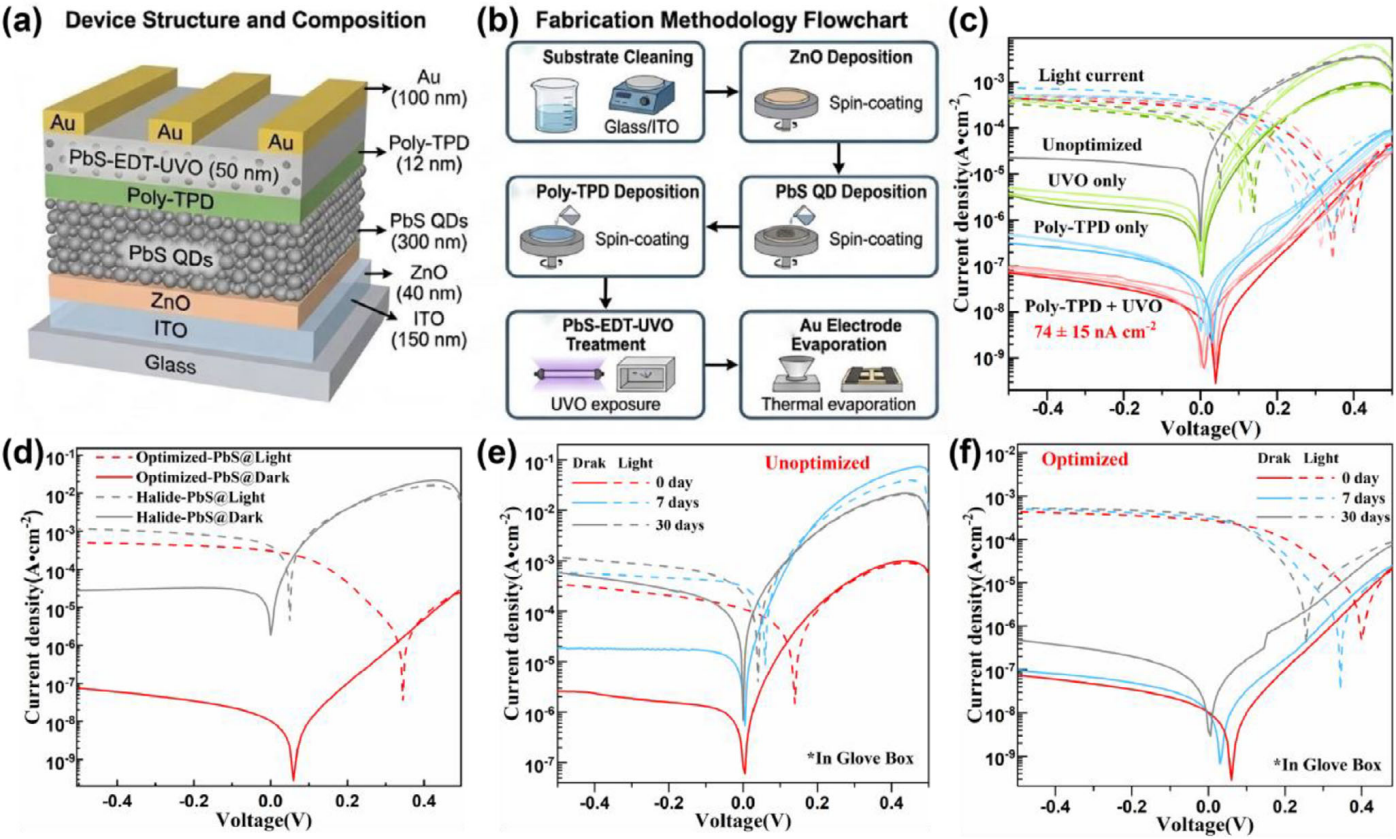
(a) Device architecture of the optimized PbS CQD photodiode. (b) Fabrication flow of the PbS CQD photodiode. (c) Factorial device‐level comparison of dark and illuminated *J–V* characteristics for unoptimized control, UVO‐only, Poly‐TPD‐only, and Poly‐TPD + UVO devices measured under identical conditions. Solid and dashed curves represent dark and 1350 nm‐illuminated currents, respectively. (d) Representative *J–V* characteristics of the unoptimized and optimized PbS CQD photodiodes measured in the dark and under 1350 nm illumination. Dark and illuminated *J–V* curves of the (e) unoptimized, and (f) optimized devices recorded after 0, 7, and 30 days of storage in a glovebox under 1350 nm NIR illumination.

The PbS CQD photodiode adopts a glass/ITO/ZnO/PbS CQDs/Poly‐TPD/PbS‐EDT‐UVO/Au stack, where a compact Poly‐TPD interlayer is inserted at the absorber/HTL interface; all layer thicknesses were determined by profilometry (Figure 1a). The fabrication sequence—including substrate cleaning, ZnO and PbS CQD deposition, Poly‐TPD coating, UVO treatment of PbS‐EDT, and Au evaporation—is summarized in Figure 1b, with full experimental details provided in the Experimental Section. To clarify the contribution of each interfacial treatment, Figure 1c presents a device‐level factorial comparison of unoptimized control, UVO only, Poly‐TPD only, and Poly‐TPD + UVO devices under identical dark and 1350 nm illumination conditions. At V = −0.5 V, the unoptimized reference device shows a high dark current density of 22568 nA cm^−^^2^. The UVO only and Poly‐TPD only devices reduce this value to 4257 ± 600 and 441 ± 50 nA cm^−^^2^, respectively, while the combined Poly‐TPD + UVO devices further suppress it to 74 ± 15 nA cm^−^^2^. Values for the engineered groups are given as mean ± s.d. from four independent devices.

This factorial comparison indicates that the two treatments contribute in a complementary manner: the Poly‐TPD interlayer mainly mitigates EDT‐induced interfacial leakage/shunt pathways, whereas UVO treatment further improves the PbS‐EDT layer by suppressing trap‐related contributions and enhancing carrier selectivity [36]. Following this comparison, Figure 1d shows representative dark and illuminated *J–V* curves of the unoptimized and optimized devices. The optimized device maintains a distinct photocurrent response while achieving nearly two orders of magnitude lower dark current than the unoptimized control. In addition, the photovoltage extracted from the illuminated *J–V* curve increases from 0.05 to 0.35 V, indicating reduced voltage loss and improved junction selectivity [37]. These results support that the dual‐interface strategy effectively suppresses dark current without compromising photocarrier extraction.

Device stability was further examined by tracking dark and illuminated *J*‐*V* curves after storage in a glovebox (H_2_O ≈ 1 ppm, O_2_ ≈ 40 ppm) for 0, 7, and 30 days. The unoptimized device (Figure 1e) shows an ∼one‐order‐of‐magnitude increase in dark current density at −0.5 V by day 7 (to 1.85×10^3^ nA cm^−^^2^), and after 30 days, the photocurrent‐dark current contrast drops below 10%, indicating near loss of effective operation. In contrast, the optimized device (Figure 1f) maintains a nearly unchanged dark current at day 7 (97 nA cm^−^^2^ at −0.5 V) and increases only slightly to 478 nA cm^−^^2^ after 30 days, remaining well below its initial photocurrent level. These results confirm that the Poly‐TPD interlayer not only improves operational stability but also mitigates moisture‐ and oxygen‐induced degradation, thereby enhancing device robustness and yield. Ambient‐air storage tests of unencapsulated devices are provided in Figure S1; the faster degradation under uncontrolled humidity/oxygen exposure highlights the need for encapsulation and controlled air‐stability evaluation.

**FIGURE 2:**
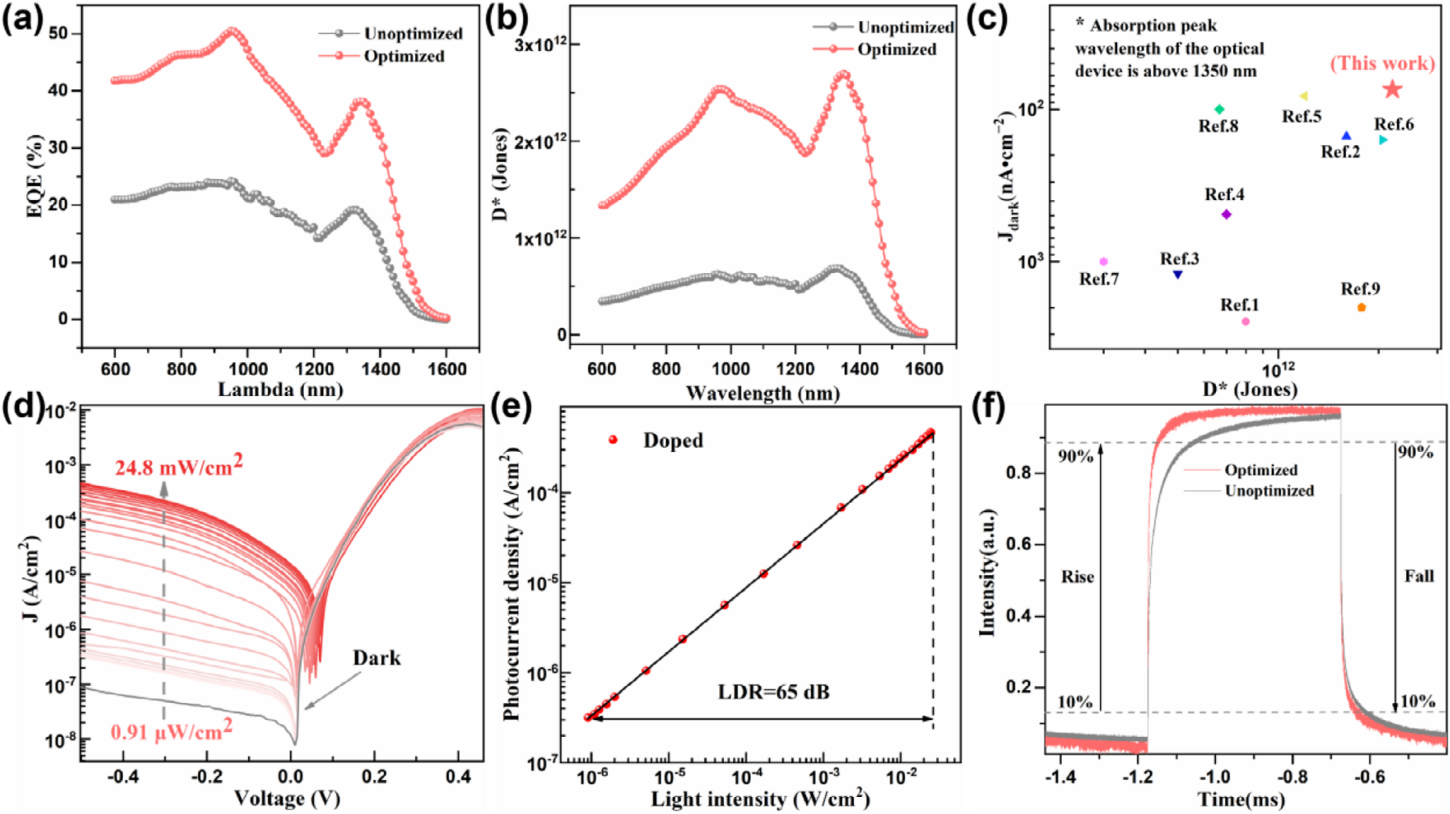
(a) EQE spectra measured under a reverse bias of −0.5 V. (b) Noise‐spectrum‐based (D^*^_meas_) spectra calculated under the same bias condition. (c) Benchmark comparison of J_dark_ and D* for reported PbS CQD photodiodes with absorption peak/cutoff wavelengths ≥1350 nm. For consistency, only J_dark_ values measured at −0.5 V are included in the main comparison; data are collected from SI‐Refs. [S1–S9] and detailed measurement conditions are summarized in Table S1. (d) Current density‐Voltage curves under dark and 1350 nm LED illumination with different power densities. (e) Linear dynamic range at bias of −0.5 V. (f) Temporal response of the PbS QD photodetector before and after optimization.

To further quantify the optical response, we measured the broadband photoresponse of the control and optimized PbS CQD devices under a −0.5 V reverse bias (Figure S2). The external quantum efficiency (EQE) describes the photon‐to‐charge conversion capability of a photodetector and is defined as the ratio of collected charge carriers to incident photons. EQE is calculated as [38]:

(1) $$\mathrm{EQE}=\frac{I_{\mathrm{ph}}/e}{P_{\mathrm{in}}/h\nu }=R\frac{\mathrm{hc}}{e\lambda }$$

where R is the responsivity, h is Planck's constant, c is the speed of light, λ is the incident wavelength, and e is the elementary charge, and the EQE spectra measured from 600 to 1600 nm under a reverse bias of −0.5 V are shown in Figure 2a. Responsivity is a key figure of merit for photodiodes, quantifying the electrical output per incident optical power. It is defined according to the equation [39]:

(2) $$R(\lambda )=\frac{\left(I_{\mathrm{ph}}-I_{\mathrm{dark}}\right)}{P_{\mathrm{in}}\;A}$$

where I_ph_ is the photocurrent, I_dark_ is the dark current, P_in_ is the incident optical power, and A is the device area (0.045 cm^2^). The optimized PbS CQD photodiode reaches R = 0.42 A W^−1^ at 1350 nm with an EQE of 38.2%, indicating efficient carrier extraction and practical NIR photoconversion at long wavelengths.

Specific detectivity (D*) was evaluated from experimentally measured dark‐current noise spectral density, Sn(f), rather than the shot‐noise‐limited approximation, providing a more realistic sensitivity benchmark. Noise spectra were measured at −0.5 V (Figure S3), where S_n_(f) is expressed in A^2^ Hz^−1^ and $i_{\mathrm{noise}}\left(f\right)=\sqrt{S_{n}\left(f\right)}$. The control device shows a relatively high, dispersed noise floor, with Sn spanning ∼10^−20^ to ∼10^−24^ A^2^ Hz^−1^ at 100 kHz. In contrast, the optimized device exhibits strongly suppressed, convergent noise spectra, reaching ∼10^−27^ to 10^−28^ A^2^ Hz^−1^ at 100 kHz. The noise‐spectrum‐based detectivity was calculated as [40]:

(3) $$D_{\mathrm{meas}}^{*}(\lambda )=R(\lambda )\frac{\sqrt{A}}{S_{n}}$$

The resulting D*_meas_ values are 6.7 × 10^11^ Jones for the control device and 2.1 × 10^12^ Jones for the optimized device at 1350 nm (100 kHz) and a reverse bias of −0.5 V. These results demonstrate that the dual‐interface strategy effectively suppresses trap‐related noise associated with carrier trapping–detrapping processes, thereby providing a more stringent and realistic sensitivity benchmark, while also improving device‐to‐device uniformity. The noise reduction is consistent with the concurrently suppressed dark current and reduced trap‐assisted injection contribution, supporting a qualitative picture in which the device transport becomes more junction‐controlled after interface optimization.

As shown in (Figure 2c), we benchmark our PbS CQD photodiode against recently reported NIR devices with an absorption peak (or cutoff response) ≥ 1350 nm, using dark current density (J_dark_) and specific detectivity (D*) as the key metrics. Our device simultaneously delivers a high D* and a markedly reduced J_dark_, placing it in the advantageous high‐detectivity/low‐dark‐current regime. Notably, under reverse bias, J_dark_ can be suppressed to 10^2^ nA cm^−2^, placing it among the lowest dark‐current values reported for long‐wavelength PbS CQD photodiodes. This ultralow dark current lowers the noise floor, thereby supporting higher D* and improved weak‐light sensing, underscoring the competitiveness of the dual‐interface strategy for long‐wavelength NIR photodiodes. The linear dynamic range (LDR) quantifies the intensity interval over which the photocurrent remains linear and is calculated as:

(4) $$\mathrm{LDR}=20\log\frac{J_{\max}}{J_{\min}}$$

where J_max_ and J_min_ are the maximum and minimum photocurrent densities before deviation from linearity. (Figure 2d) shows the J‐V curves measured at 1350 nm under varying power densities: in the dark, the reverse‐bias current remains low and stable; with increasing illumination, the reverse photocurrent shifts upward and tends to saturate at higher reverse bias, indicating more complete carrier collection. Consistently, at V = −0.5 V, the photocurrent density scales linearly with incident intensity from 0.91 µW cm^−^^2^ to 24.8 mW cm^−^^2^ (Figure 2e), spanning nearly four decades and yielding an LDR of 65 dB, highlighting the benefit of low‐dark‐current/low‐noise transport for wide‐dynamic‐range weak‐light detection.

The temporal response of a photodetector reflects how rapidly it follows optical modulation, typically quantified by the rise and fall times. As shown in (Figure 2f), the optimized device exhibits markedly faster dynamics with rise/fall times of 17.4 µs/19.5 µs and reduced transient lag, compared with 64.6 µs/57.7 µs for the control. This acceleration is attributed to improved PbS‐halide/PbS‐EDT interfacial boundary conditions and more favorable energy‐level alignment, which together promote more efficient carrier extraction.

**FIGURE 3:**
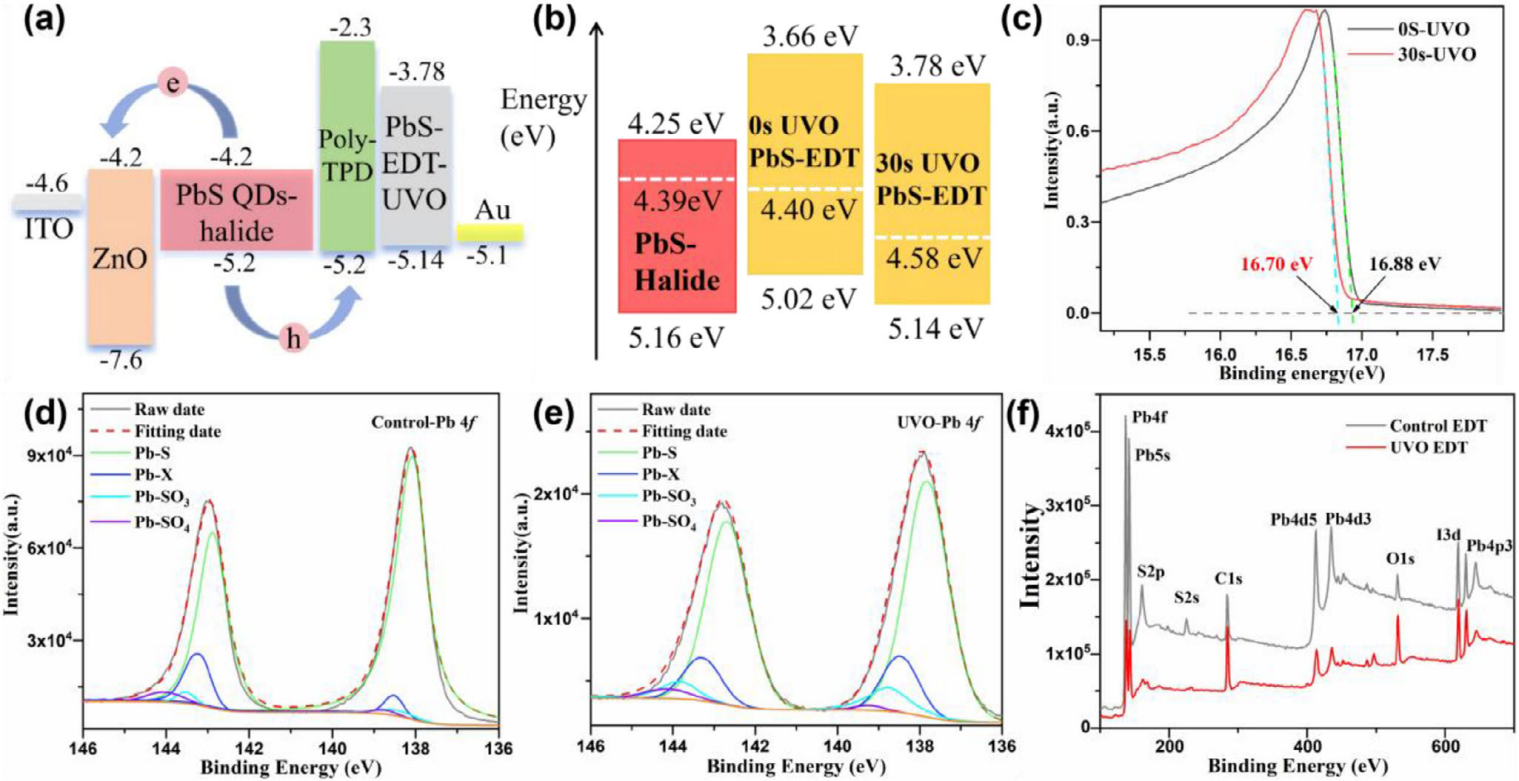
(a) Energy‐band diagram of the optimized device stack. (b) Energy‐level alignment of the PbS‐halide layer and the PbS‐EDT layer before/after UVO treatment. (c) Enlarged UPS secondary electron cutoff (SEC) region (extracted from the full UPS spectra in Figure S5), highlighting the UVO‐induced work‐function change of the PbS CQD film. (d, e) Pb 4f XPS spectra of PbS‐EDT CQDs without and with UVO oxidation (X = OH, RCOO). (f) XPS elemental composition summary.

To rationalize the enhanced interfacial selectivity and suppressed reverse injection enabled by our dual‐interface engineering, we analyze the energy‐level alignment of the optimized PbS CQD photodetector, as schematically summarized in Figure 3a. The optical properties and bandgaps of the PbS–halide and PbS–EDT films were extracted from UV–vis–NIR spectroscopy (Figure S4). Energy levels before and after treatments were further determined by UPS measurements (Figure S5), where the valence‐band maximum (VBM) and conduction‐band minimum (CBM) were derived using the Tauc‐plot approach [41]. The Poly‐TPD buffer exhibits a VBM of 5.2 eV, closely matching those of PbS‐halide (VBM = 5.2 eV) and PbS‐EDT (VBM = 5.14 eV), which facilitates efficient hole extraction and transport [15, 42, 43]. Meanwhile, its CBM (2.3 eV) lies substantially lower than those of the PbS‐halide and PbS‐EDT layers, providing an effective electron‐blocking barrier that suppresses electron injection.

UVO treatment induces a clear shift in the electronic structure of the PbS–EDT layer, as evidenced by the energy‐level comparison in Figure 3b. The UVO exposure time was optimized in Poly‐TPD‐buffered devices by comparing the dark *J–V* characteristics after 10 s, 30 s, 1 min, and 2 min treatments; 30 s most effectively suppressed the dark current and was therefore selected as the optimized condition (Figure S6). Specifically, the Fermi level shifts to a deeper energy level (more negative with respect to vacuum), consistent with an increase in p‐type character. Importantly, the treated PbS–EDT maintains a VBM of 5.14 eV, which closely matches that of PbS–halide (VBM = 5.16 eV) and Poly‐TPD (VBM = 5.2 eV), resulting in a small valence‐band offset (ΔE_VBM_ < 0.06 eV) that facilitates hole extraction [22]. The UPS secondary electron cutoff (SEC) region is further enlarged in Figure 3c; SEC analysis yields work functions (relative to vacuum) of 4.40 eV for pristine PbS–EDT and 4.58 eV for the 30 s UVO‐treated film (PbS–EDT–30 s), corresponding to a 0.18 eV shift upon UVO exposure.

XPS examined surface chemical changes induced by UVO, as shown by the Pb 4f spectra (Figure 3d,e). The Pb‐S peaks at 138.06 and 142.88 eV decrease, whereas the Pb‐SO_3_ (138.60 and 143.52 eV) and Pb‐SO_4_ (138.76 and 144.08 eV) components increase, consistent with oxidation signatures reported for PbS QD films [30]. In addition, the elemental composition (Figure 3f) shows a marked increase in the O1s atomic fraction (12.3% → 23.7%) accompanied by decreases in the S2p and P2p signals, confirming oxide‐assisted passivation of thiol‐capped PbS‐EDT and the concomitant enhancement of p‐type doping.

**FIGURE 4:**
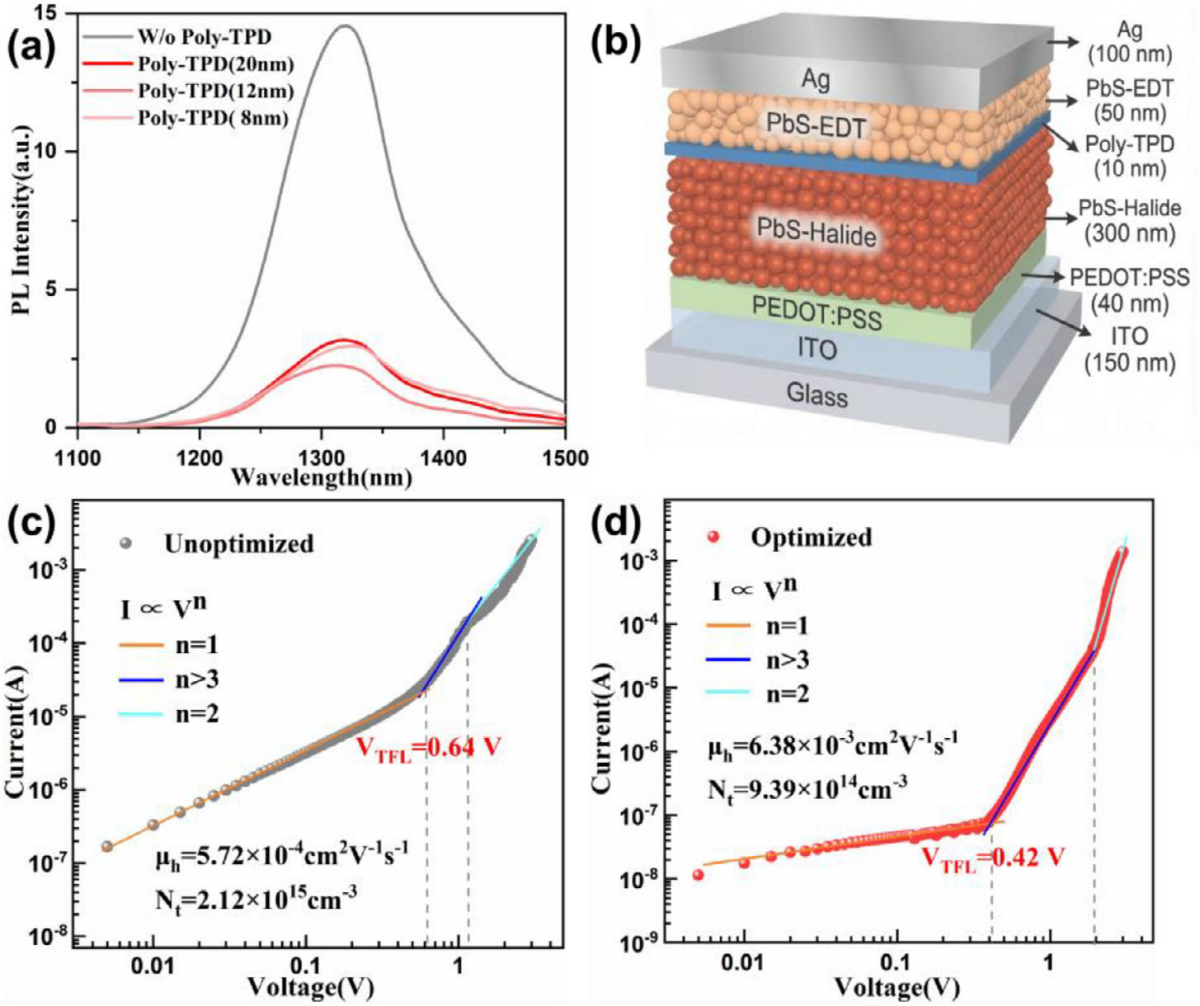
(a) PL spectra of PbS‐halide films without Poly‐TPD and with Poly‐TPD interlayers of different thicknesses. (b) Hole‐only device structure for single‐carrier transport. (c, d) SCLC analysis of hole transport, with Ohmic (n = 1), trap‐filled limit (n > 3), and Child's (n = 2) regimes indicated; V_TFL_ denotes the trap‐filled‐limit voltage. The µ_h_ and N_t_ were extracted by fitting the I‐V characteristics.

To probe interfacial carrier extraction at the PbS‐halide/Poly‐TPD heterojunction, PL measurements were performed on bilayer stacks with different Poly‐TPD thicknesses (Figure 4a). All Poly‐TPD‐containing stacks exhibit pronounced PL quenching relative to pristine PbS‐halide, indicating suppressed radiative recombination and efficient interfacial charge transfer, consistent with the reported correlation between PL quenching and improved charge extraction [44]. Thickness‐dependent device measurements further reveal a clear trade‐off: the ∼12 nm Poly‐TPD layer, obtained from a 5 mg mL^−^^1^ solution, gives the lowest dark current, whereas thinner (∼3 nm) or thicker (∼20 nm) interlayers show less favorable current characteristics (Figure S7; thickness profiles in Figure S8). These results suggest that the optimized Poly‐TPD thickness balances interfacial protection and hole transport.

To corroborate improved hole extraction, we further analyzed the dark *J–V* characteristics of a hole‐only device (ITO/PEDOT:PSS/PbS‐halide/Poly‐TPD/PbS‐EDT/Ag) using space‐charge‐limited current (SCLC) measurements (Figure 4b). Following the procedure reported by Zhu et al. [45]., the dark *J–V* curves before and after optimization are shown in Figure 4c,d. Within the SCLC framework, the hole mobility (µ_h_) is calculated as:

(5) $$J=\frac{9\varepsilon_{0}\varepsilon_{r}\mu_{0}V^{2}}{8L^{3}}$$

where J is the current density, µ_0_ is the zero‐field mobility, ε_0_ is the vacuum permittivity, ε_r_ is the relative permittivity, V is the effective voltage, and L is the active layer thickness. The mobility can be calculated as [46]:

(6) $$\mu =\frac{8L^{3}}{9\varepsilon_{0}\varepsilon_{r}}\;\frac{\partial J}{\partial \left(V^{2}\right)}$$

Using the measured thickness (L) (profilometry; Figure S8), we obtain µ_h_ = 5.72 × 10^−4^ cm^2^ V^−1^ s^−1^ and 6.38 × 10^−3^ cm^2^ V^−1^ s^−1^ for the control and optimized devices, respectively, corresponding to an ∼11.1× enhancement and indicating more efficient hole transport for improved carrier extraction. With the Poly‐TPD interlayer, the hole‐only device exhibits a lower trap‐filled‐limit voltage (V_FTL_ = 0.42 V) than the control (V_FTL_ = 0.64 V). The trap density (N_t_) is calculated as [46]:

(7) $$N_{t}=\frac{2V_{\mathrm{TFL}}\varepsilon_{0}\varepsilon_{r}}{eL^{2}}$$

where ε_r_ is the relative permittivity, ε_0_ is the vacuum permittivity, e is the elementary charge, L is the active‐layer thickness, and V_FTL_ is the trap‐filled‐limit voltage. In PbS CQD photodiodes, the trap density (N_t_) is a key defect metric that reflects passivation quality and interfacial defect levels. From Equation (7), N_t_ decreases from 2.12 × 10^15^ to 9.39 × 10^14^ cm^−3^ after optimization (>50% reduction), which suppresses trap‐assisted injection and thereby lowers the dark current (2.768 × 10^4^ → 74 nA cm^−^^2^), leading to enhanced responsivity and D*. It should be noted that the extracted µ_h_ and N_t_ are effective parameters of the hole‐only multilayer device, mainly reflecting hole transport and trap filling across the PbS‐halide/Poly‐TPD/PbS‐EDT interfacial transport region, rather than the intrinsic mobility or trap density of an individual isolated layer.

**FIGURE 5:**
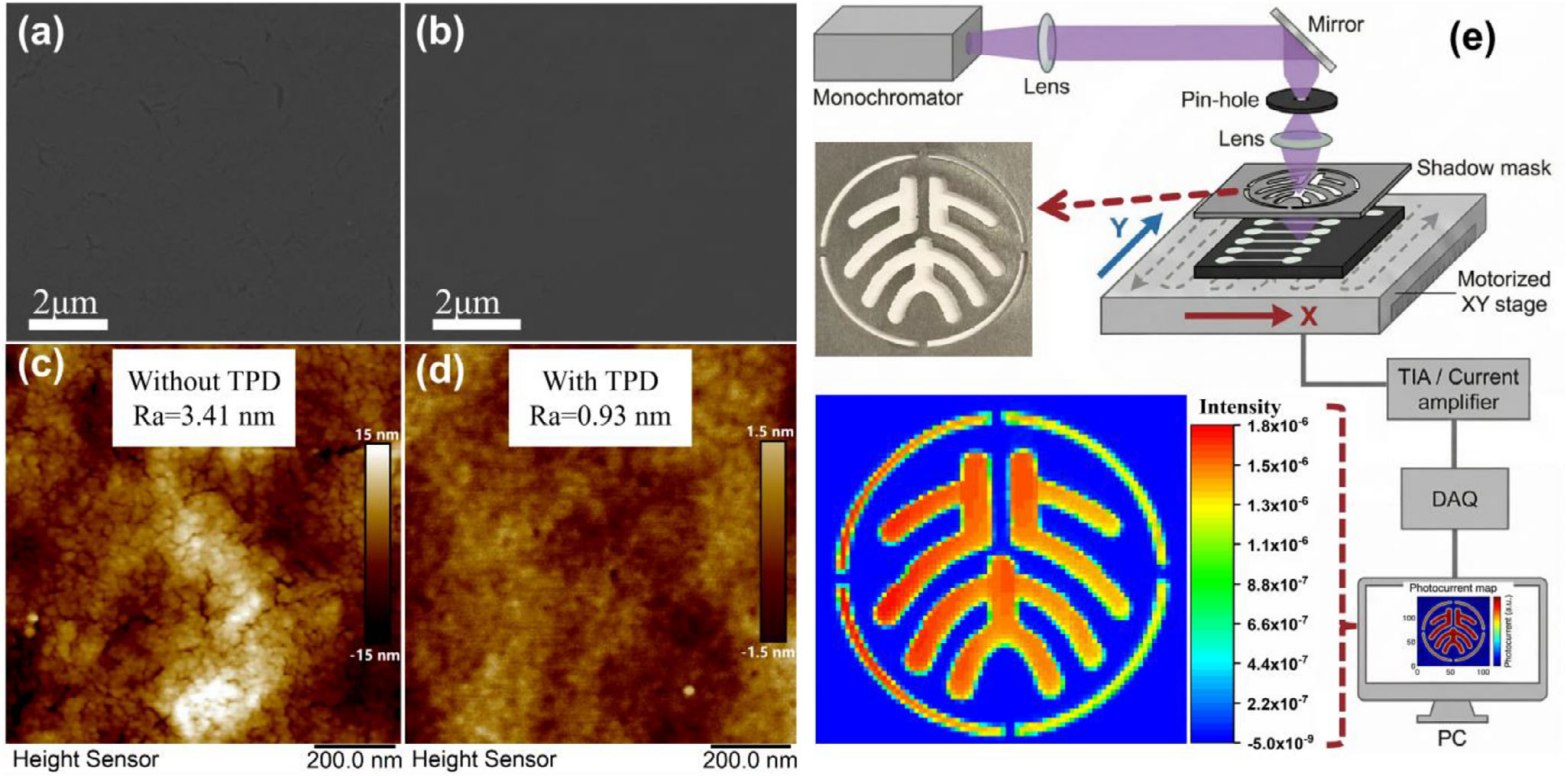
SEM images of PbS‐halide films after EDT processing (a) without and (b) with a Poly‐TPD interlayer. AFM topography of films (c) without TPD (Ra = 3.41 nm) and (d) with TPD (Ra = 0.93 nm). (e) Point‐by‐point raster‐scan transmission imaging using the PbS CQD photodiode: monochromated NIR light (λ = 1250–1400 nm) is pinhole‐filtered and focused onto a metal shadow mask (Peking University emblem) above the device; a motorized XY scan samples the transmitted signal (step 40 µm), amplified (TIA), digitized (DAQ), and reconstructed into a 2D photocurrent map at 1.2 mW cm^−^^2^ under zero‐bias (photovoltaic) operation. (The Peking University school mark /emblem is used with permission from Peking University).

To elucidate the protective role of the Poly‐TPD interlayer, we characterized the stacks by SEM and AFM after mimicking EDT processing. PbS‐halide films with and without Poly‐TPD were treated with 0.02% EDT in acetonitrile for 2 mins and then rinsed twice with acetonitrile. SEM reveals pronounced micron‐scale cracks on PbS‐halide without Poly‐TPD (Figure 5a), which can act as leakage/short‐circuit pathways [47], thereby increasing dark current [48] and reducing yield; AFM confirms a high roughness (Ra = 3.41 nm, Figure 5c). By contrast, introducing Poly‐TPD markedly improves the surface morphology (Figure 5b), with substantially fewer cracks and a smoother interface, and reduces roughness to Ra = 0.93 nm (Figure 5d). Such a smooth interface is known to facilitate carrier extraction in diode devices [49, 50], consistent with the performance improvement observed in Figure 1c. Additional XPS measurements were performed on EDT‐treated PbS‐halide films. The exposed PbS‐halide film without Poly‐TPD shows clear Pb, S, I, and Br signals in the XPS survey spectrum (Figure S9). However, for the Poly‐TPD‐covered PbS‐halide film, the signal is dominated by the surface Poly‐TPD layer because the optimized Poly‐TPD thickness (∼12 nm) exceeds the effective probing depth of conventional XPS. Thus, direct chemical quantification of the buried PbS‐halide layer is not reliable in this stacked structure. Therefore, the protective effect of Poly‐TPD is primarily supported by the combined SEM/AFM morphology, improved device statistics, and suppressed leakage current.

To directly demonstrate the feasibility of NIR imaging, we built a transmission imaging platform based on single‐pixel raster scanning (Figure 5e). Monochromated light (λ = 1250–1400 nm) was collimated/focused, folded by a mirror, and spatially filtered by a pinhole to form a stable narrow beam, which illuminated a metal shadow mask patterned with the Peking University emblem at an incident power density of 1.2 mW cm^−^^2^. The device, mounted on a motorized XY stage, was raster‐scanned with a 40 µm step to sample the transmitted intensity; signals pass through in the apertures and are strongly suppressed in the opaque regions. At each pixel, the photocurrent was amplified by a transimpedance amplifier (TIA), recorded by a data‐acquisition (DAQ) unit, and reconstructed into a 2D photocurrent map. The reconstructed image clearly reproduces the mask outline and internal features, indicating good spatial response uniformity and discernible contrast under a tightly focused beam. Notably, under zero‐bias (photovoltaic) operation, the image quality is largely limited by the noise floor; the ultralow dark current density of our device suppresses the background current and its associated noise, maintaining precise edges and pattern fidelity under NIR illumination. This observation is consistent with the suppressed dark current and low‐noise transport enabled by dual‐interface engineering, highlighting its potential for weak‐light NIR imaging.

## 3 Overall Discussion

**FIGURE 6:**
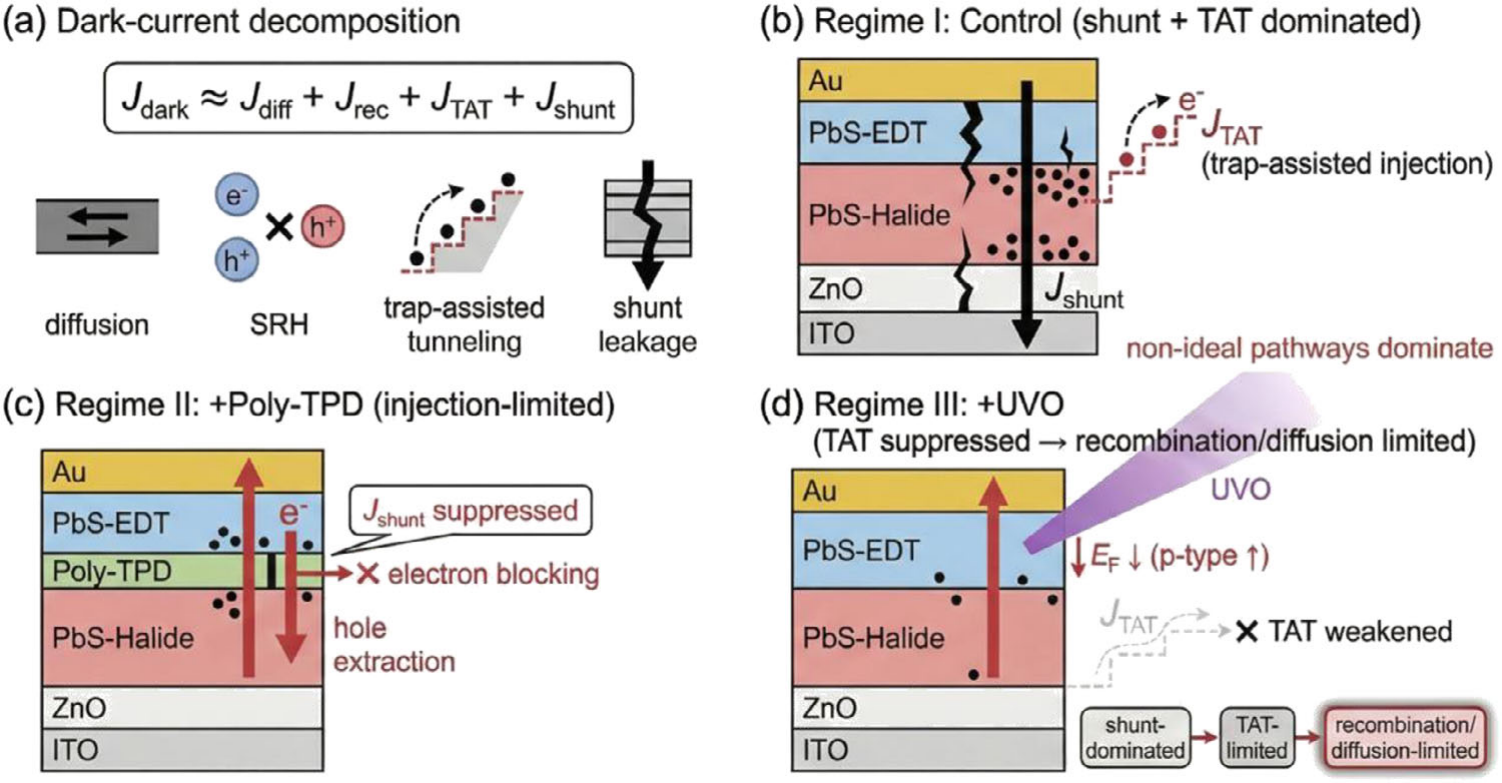
Qualitative interpretation of synergistic dual‐interface engineering. (a) Possible reverse‐bias dark‐current pathways in PbS CQD photodiodes, including diffusion, SRH recombination, trap‐assisted tunneling/injection, and shunt leakage. (b) In the control device, interfacial roughening and trap states may promote shunt leakage and trap‐assisted injection. (c) The Poly‐TPD buffer is expected to improve interfacial continuity, reduce leakage pathways, and provide electron blocking. (d) UVO‐treated PbS‐EDT may reduce trap‐related contributions and improve carrier selectivity, resulting in lower dark current and more stable, low‐noise diode operation.

The reverse‐bias dark current in PbS CQD photodiodes can be qualitatively discussed by considering several possible contributions, including quasi‐neutral diffusion, depletion‐region Shockley–Read–Hall recombination, trap‐assisted tunneling/injection, and shunt leakage from film discontinuities or pinholes (Figure 6a) [49, 50]:

(8) $$J_{\mathrm{dark}}\;\approx \;J_{\mathrm{diff}}+J_{\mathrm{rec}}+J_{\mathrm{TAT}}+J_{\mathrm{shunt}}$$

here, Equation (8) is used as a phenomenological framework rather than a quantitative decomposition of the individual current components. In the control device, EDT/acetonitrile processing may roughen the PbS‐halide/PbS‐EDT interface and generate microcracks, thereby promoting shunt‐related leakage [50]. Meanwhile, interfacial trap states can provide intermediate pathways for trap‐assisted injection, contributing to non‐ideal reverse‐bias transport(Figure 6b) [50, 51]. The Poly‐TPD interlayer is expected to improve interfacial continuity and reduce crack‐/pinhole‐mediated leakage, while its conduction‐band offset provides an electron‐blocking boundary that may suppress reverse electron injection(Figure 6c) [50, 52]. Subsequent UVO treatment of PbS‐EDT further modifies the interfacial electronic structure by enhancing p‐type character and reducing the effective trap density, which is consistent with weakened trap‐related injection and reduced noise(Figure 6d) [50, 53].

Overall, the combined morphological, energetic, SCLC, and noise‐spectral evidence supports a qualitative picture in which Poly‐TPD mainly mitigates leakage‐related pathways, whereas UVO further suppresses trap‐related contributions [54]. This interpretation rationalizes the simultaneous reduction of dark current and noise without sacrificing carrier extraction. However, a rigorous quantitative separation of J_diff_, J_rec_, J_TAT_, and J_shunt_ would require further dedicated transport measurements or numerical simulations.

## 4 Conclusion

The dual‐interface engineering strategy reported here resolves key interfacial limitations in PbS CQD photodetectors. By inserting a Poly‐TPD buffer at the absorber/HTL junction and applying controlled UVO oxidation to the HTL, we simultaneously mitigate EDT‐induced interfacial damage, enhance p‐type character, and passivate deep defects. The synergy reduces the trap density by ∼55% (2.12 × 10^15^ cm^−3^→9.39 × 10^14^ cm^−3^) and boosts the hole mobility by ∼11× (5.72 × 10^−4^ cm^2^ V^−1^ s^−1^ → 6.38 × 10^−3^ cm^2^ V^−1^ s^−1^). Consequently, the dark current density decreases to 74 nA cm^−2^ at ‐0.5 V (≈2 orders of magnitude reduction), while D* reaches 2.1 × 10^12^ Jones and the rise/fall times shorten to 17.4/19.5 µs. These results place the device among the higher‐performing solution‐processed PbS CQD photodiodes for 1.3–1.4 µm NIR detection, while further improvements in array uniformity, bandwidth, and long‐term operational stability will be needed for direct comparison with commercial InGaAs technologies. This simple, scalable interface paradigm provides a practical route toward low‐cost, high‐sensitivity NIR photodetectors, with potential to accelerate applications in biosensing, optical communications, and related fields.

## 5 Experimental Section

This section summarizes the synthesis of PbS CQDs and ZnO nanocrystals, the ligand‐exchange protocol, and key device processing conditions to ensure reproducibility and parameter traceability.

### 5.1 Synthesis of PbS QDs and Ligand Exchange

PbS CQDs for the HTL were synthesized by a rapid hot‐injection method [55], yielding a first excitonic peak at ∼950 nm. PbS CQDs for the absorber (first excitonic peak at ∼1350 nm; TEM and UV–vis‐NIR spectra in Figure S10) were prepared via a cation‐exchange route [56]. Ligand exchange was performed in air, following the Sargent group's protocol [57]. The exchange solution was prepared by dissolving 0.366 M PbI_2_, 0.036 M PbBr_2_, 0.2 M KI, and 0.01 M ammonium acetate in N,N‐dimethylformamide (DMF). A hexane dispersion of PbS CQDs (10 mg mL^−^^1^; ∼1350 nm first excitonic peak) was mixed with the exchange solution at a 1:1 volume ratio and shaken until complete phase transfer into the DMF phase; the upper hexane layer was then removed. The DMF phase was washed three times with hexane as the antisolvent. After exchange, toluene was added as an antisolvent, followed by centrifugation; the precipitate was vacuum‐dried. The halide‐capped PbS CQDs for absorber deposition were re‐dispersed in a BTA/DMF mixed solvent (v/v = 4:1) at 320 mg mL^−^^1^.

### 5.2 Synthesis of ZnO Nanocrystals

ZnO nanocrystal sol was synthesized following the method of Bawendi et al. [15]. by dissolving ethanolamine (95 µL) and zinc acetate (0.3 g) in 2 mL 2‐methoxyethanol and stirring at 60°C for 24 h.

### 5.3 Device Fabrication

ITO substrates were ultrasonically cleaned in acetone, isopropanol, and deionized water to remove surface residues. The PbS‐halide absorber was then spin‐coated onto the ZnO layer as described above. A dilute Poly‐TPD buffer was deposited on PbS‐halide by spin‐coating a 3–5 mg mL^−^^1^ chlorobenzene solution at 2000 rpm for 20 s, followed by annealing at 70°C for 10 min in an inert glovebox. Next, two PbS‐EDT layers were deposited as the hole‐extraction layer: for each layer, 50 µL of a 45 mg mL^−^^1^ PbS CQD hexane solution was spin‐coated (2000 rpm, 20 s), treated with 0.02 vol% EDT in acetonitrile for 2 min, and rinsed twice with acetonitrile; the film was then annealed at 80°C in the glovebox. The surface was subsequently subjected to UV‐ozone treatment (10 s‐2 min) using a commercial 254 nm Hg lamp. Finally, a 100 nm Au top electrode was deposited by thermal evaporation. The effective device area was 0.045 cm^2^.

SEM images were obtained using an FEI Nova Nano SEM 450. The Spectra 300 Condenser Lens Cs‐corrected TEM (Thermo Fisher Scientific, USA) was used in scanning TEM (STEM) mode, while in high‐resolution TEM (HRTEM) mode, the samples were observed using the Spectra 300 Objective Lens Cs‐corrected TEM (Thermo Fisher Scientific, USA). The UV–vis‐NIR spectra were measured using a PerkinElmer Lambda 750 UV/VIS/NIR Spectrometer. The UV–vis absorption spectrum (300–1000 nm) was collected using a PerkinElmer Lambda 750 UV–vis/NIR Spectrometer. The morphology of the film was measured by atomic force microscopy (AFM) (Bruker, Multi‐Mode 8‐HR). Ultraviolet photoelectron spectroscopy (UPS) experiments were performed using a x‐ray Photoelectron Spectrometer (XPS) Microprobe (Thermo Scientific, Escalab 250Xi). A He I (21.22 eV) discharge lamp (energy resolution of 0.1 eV) was used for UPS experiments, respectively. The samples were all biased by‐10 V for better measurement of the secondary electron cutoffs.

### 5.4 Device Characterization

The current‐voltage (*I–V*) characteristics were measured using a Keithley 1500 source meter. Light intensity was calibrated using a THORLABS PM100D optical power meter. The photo‐response spectra of the QPDs were measured using a SC‐PRO super‐continuum source and a LE‐SP‐150MA monochromator. The intensity of the NIR and visible light sources was controlled using a set of neutral optical filters. The PL spectra were collected using a HORIBA modular scientific research‐grade fluorescence spectrometer. The transient response time was measured on an oscilloscope instrument (Agilent DSOS054A) with a 532 nm laser source. The noise power spectra were obtained with a preamplifier (Stanford Research Systems, SR570). The noise current spectra were recorded on a dynamic signal analyzer (Agilent 35670A).

### 5.5 Imaging Sensing Process

A transmission imaging setup based on single‐pixel raster scanning was used to evaluate the NIR imaging capability of the PbS CQD photodiode. Monochromated light (λ = 1250–1400 nm) was collimated, redirected by a mirror, and spatially filtered through a pinhole to form a stable focused beam. The beam was incident on a metal shadow mask patterned with the Peking University emblem at an optical power density of 1.2 mW cm^−2^. The device was mounted on a motorized XY stage and scanned with a step size of 40 µm to sample the transmitted intensity point by point. At each pixel, the generated photocurrent was amplified by a transimpedance amplifier (TIA), collected by a data‐acquisition (DAQ) system, and reconstructed into a two‐dimensional photocurrent map. All measurements were performed under zero‐bias (photovoltaic) operation.

## Author Contributions

**Chuan Wei**: conceptualization, investigation, funding acquisition, writing – original draft, writing – review and editing, visualization, validation, methodology, formal analysis, data curation. **Jun Han**: conceptualization, visualization, methodology, formal analysis, resources, supervision, data curation, project administration, software. **Ning Feng**: project administration, supervision, visualization. Li Yan: investigation. **Hao Sun**: investigation. **Yuanhong Gao**: funding acquisition. **Meili Xu**: methodology. **Guodan Wei**: resources. Hong Meng: resources, supervision, data curation, project administration, formal analysis, writing – review and editing.

## Conflicts of Interest

The authors declare no conflicts of interest.

## Supporting information




**Supporting File**: advs76271‐sup‐0001‐SuppMat.pdf.

## Data Availability

The data that support the findings of this study are available from the corresponding author upon reasonable request.
