# Supplementary material for: Synergistic Interface Engineering via Buffer Layer and UVO Treatment for High‐Performance PbS Quantum Dot Near‐Infrared Photodiodes
Source: Adv Sci (Weinh). 2026 Jun 25;13(52):e76271. doi: 10.1002/advs.76271 (PMC13336795; doi:10.1002/advs.76271)
Supplement: Supplementary file 1 [file ADVS-13-e76271-s001.pdf]

Supporting Information

Synergistic Interface Engineering via Buffer Layer and UVO Treatment for High-Performance PbS Quantum Dot Near-Infrared Photodiodes

Chuan Wei, Jun Han*, Ning Feng, Li Yan, Hao Sun, Yuanhong Gao, Meili Xu[†], Guodan Wei[††], and Hong Meng*

School of Advanced Materials, Shenzhen Graduate School, Peking University, Shenzhen 518055, PR China

[†]School of Flexible Electronics (SoFE), Sun Yat-sen University, Gongchang Road, Shenzhen, Guangdong 518107, China

[††]Institute of Materials Research, Shenzhen Geim Graphene Center, Shenzhen International Graduate School, Tsinghua University, Shenzhen 518055, China

**E-mail:* junhan@pku.edu.cn (J.H.) ; menghong@pku.edu.cn (H.M.)

Figures S1–S10

Tables S1

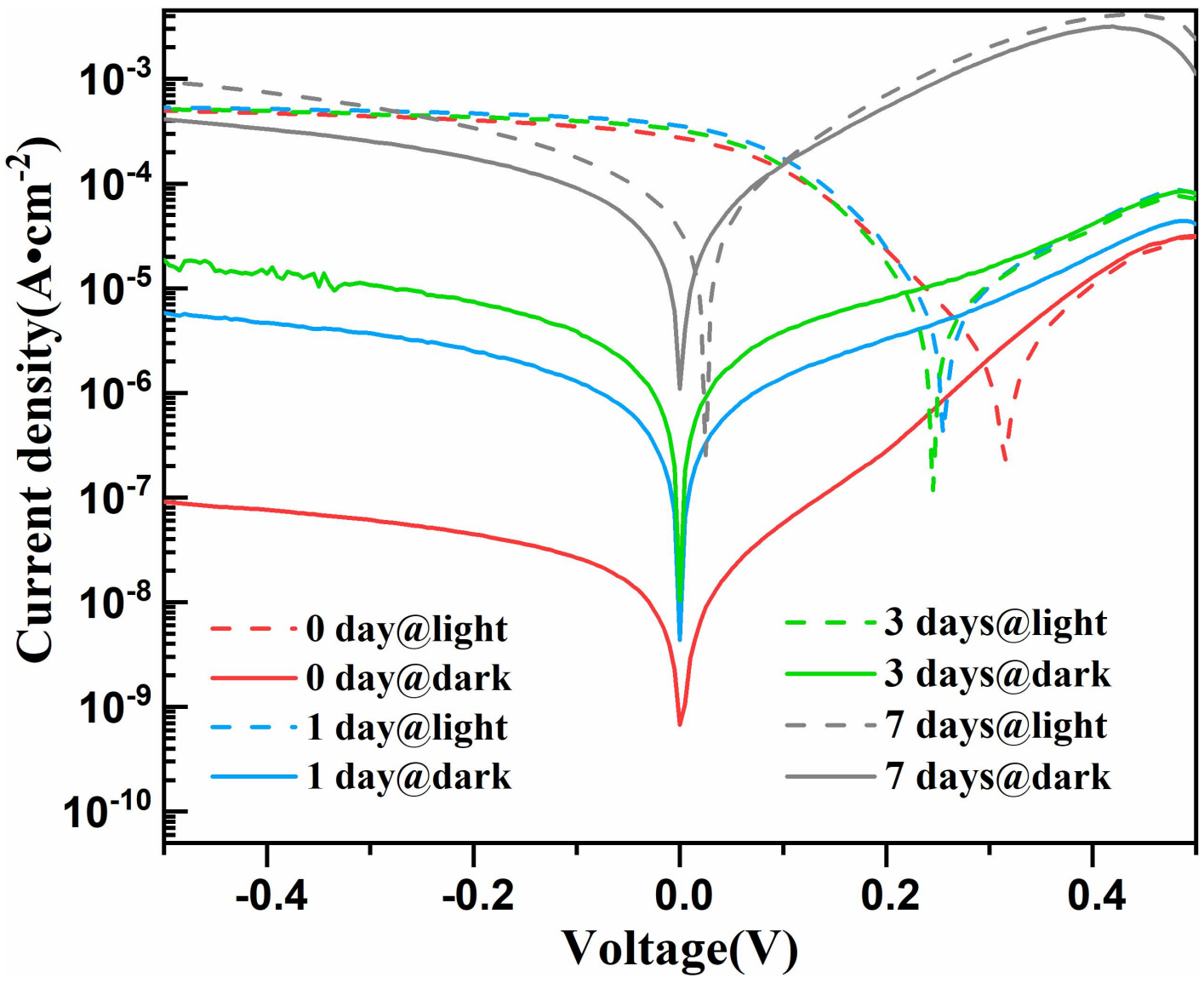

Figure S1. Ambient-air stability of unencapsulated PbS CQD photodiodes. Representative dark and illuminated J–V curves of devices stored under ambient air. The unencapsulated devices degrade substantially faster than those stored in a glovebox, with larger device-to-device variation, indicating the need for encapsulation and controlled environmental testing for practical air operation.

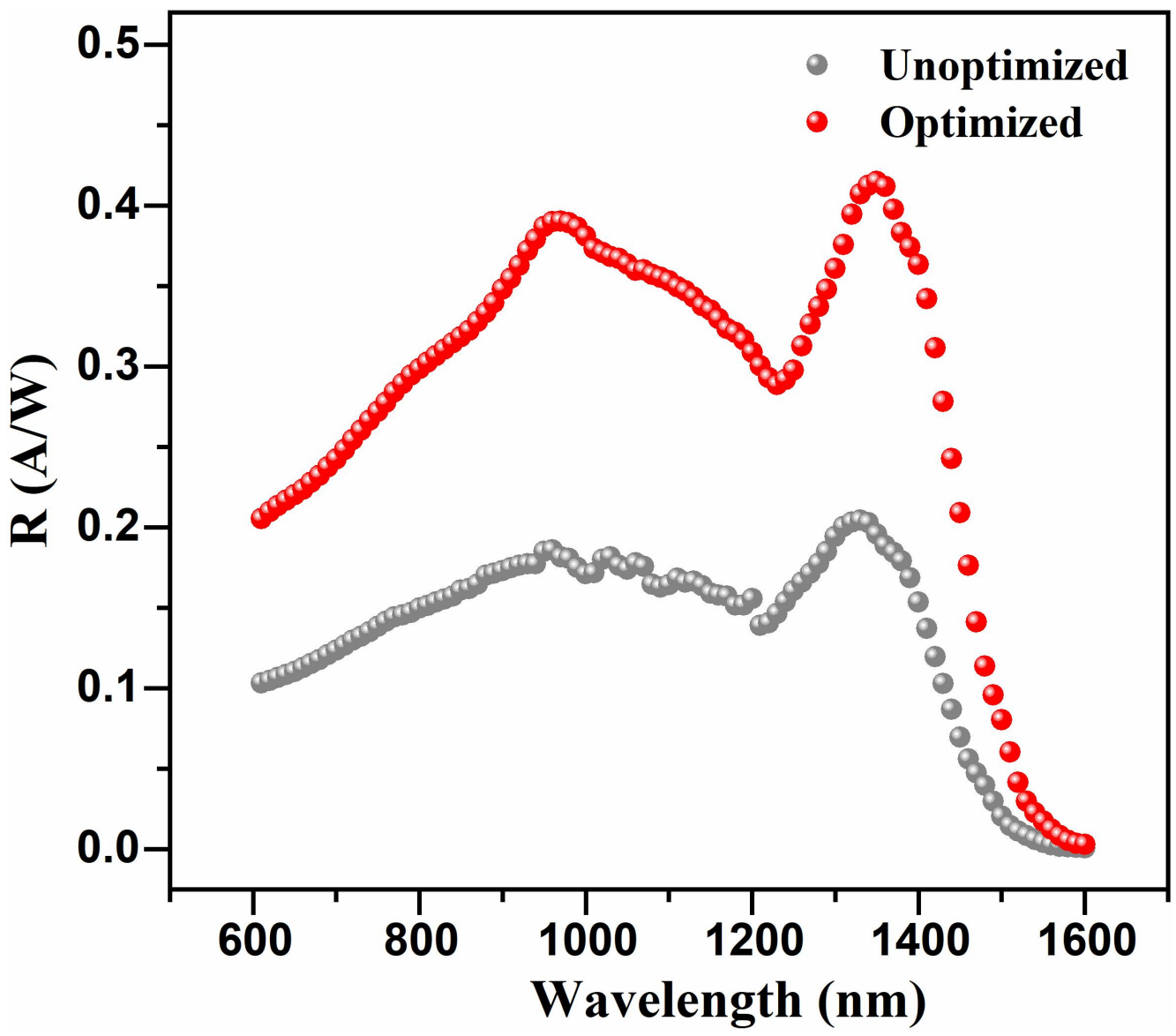

Figure S2. Responsivity spectra of the control and optimized PbS CQD devices under a reverse bias of −0.5 V.

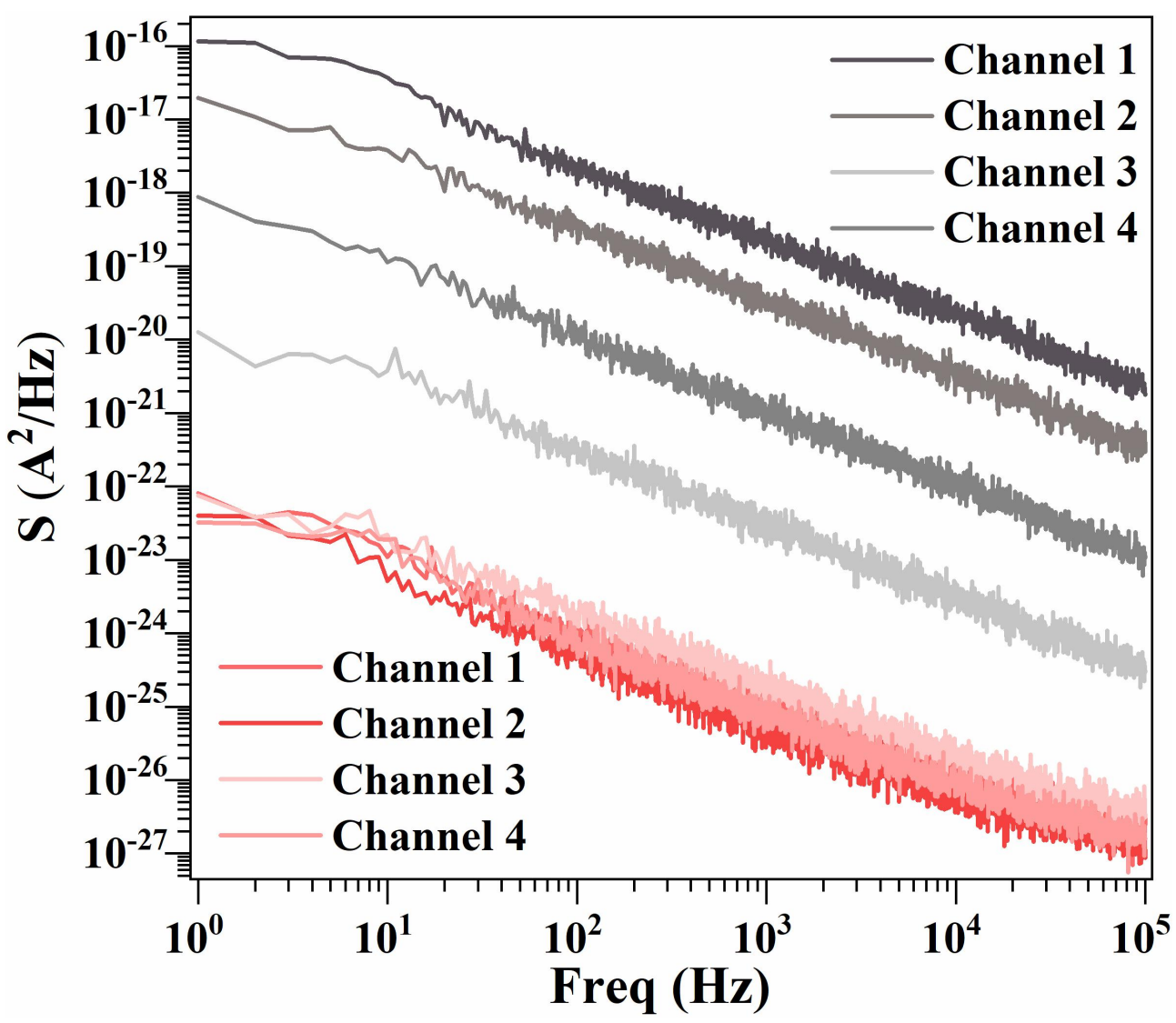

Figure S3. Noise current spectral density of four representative channels/devices before and after optimization, measured at −0.5 V.

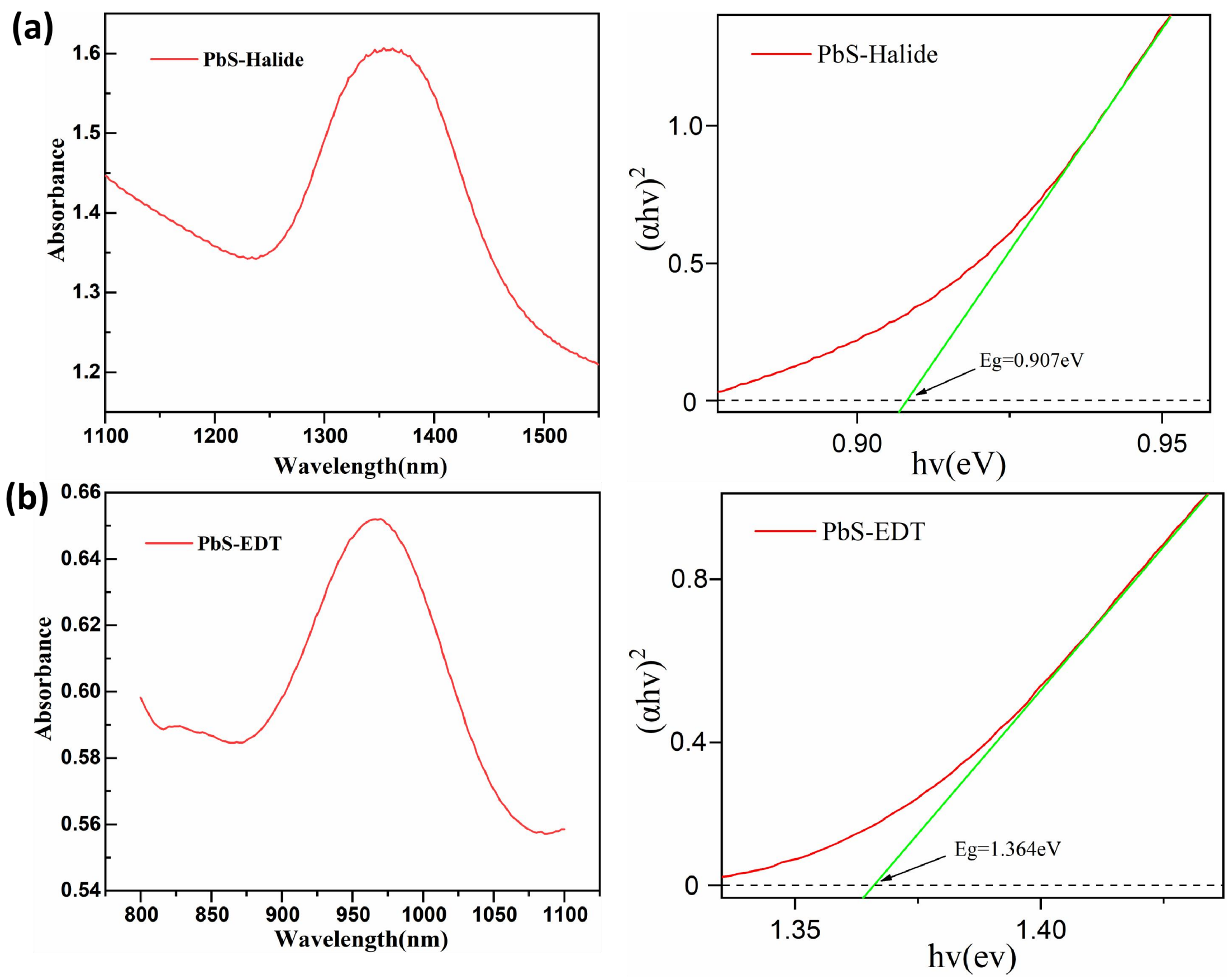

Figure S4. UV–vis–NIR absorption spectra of the (a) PbS-halide and (b) PbS-EDT layers and their corresponding bandgap energies.

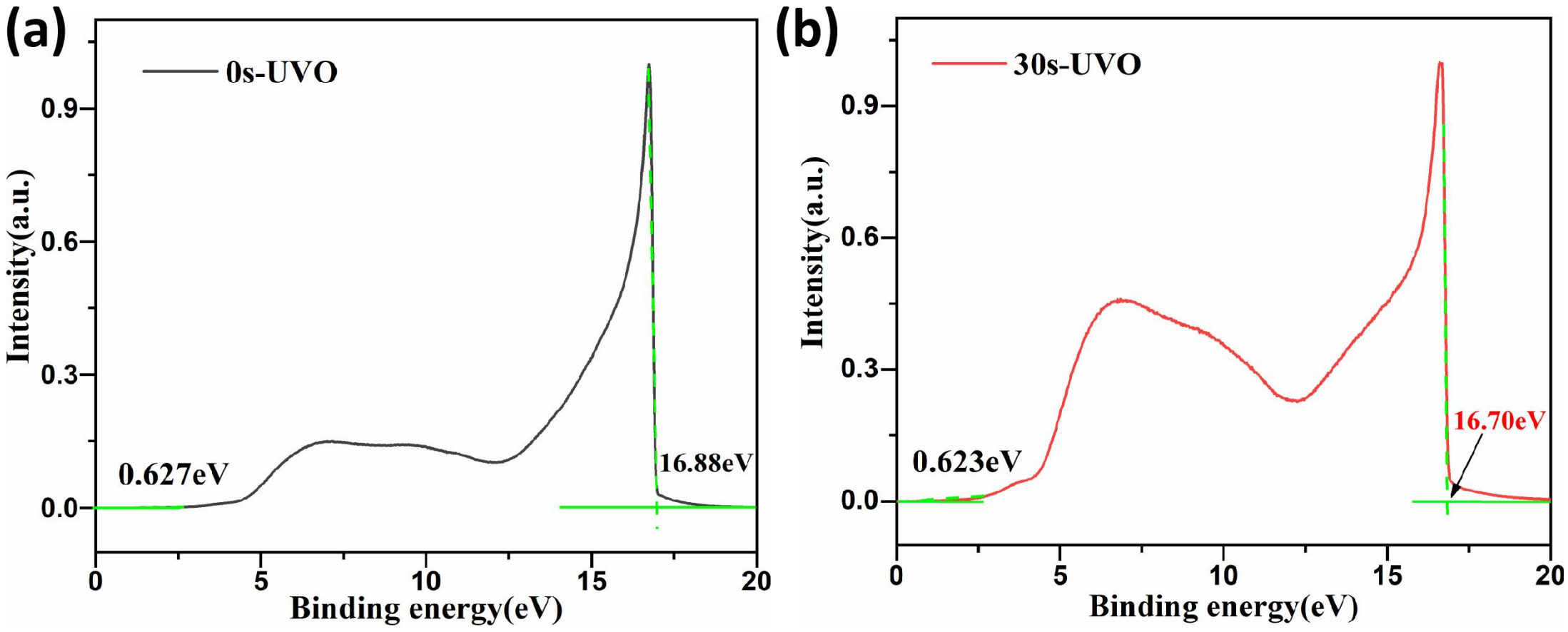

Figure S5. UPS spectra of PbS-EDT CQDs without (a) and with (b) UVO Treatment

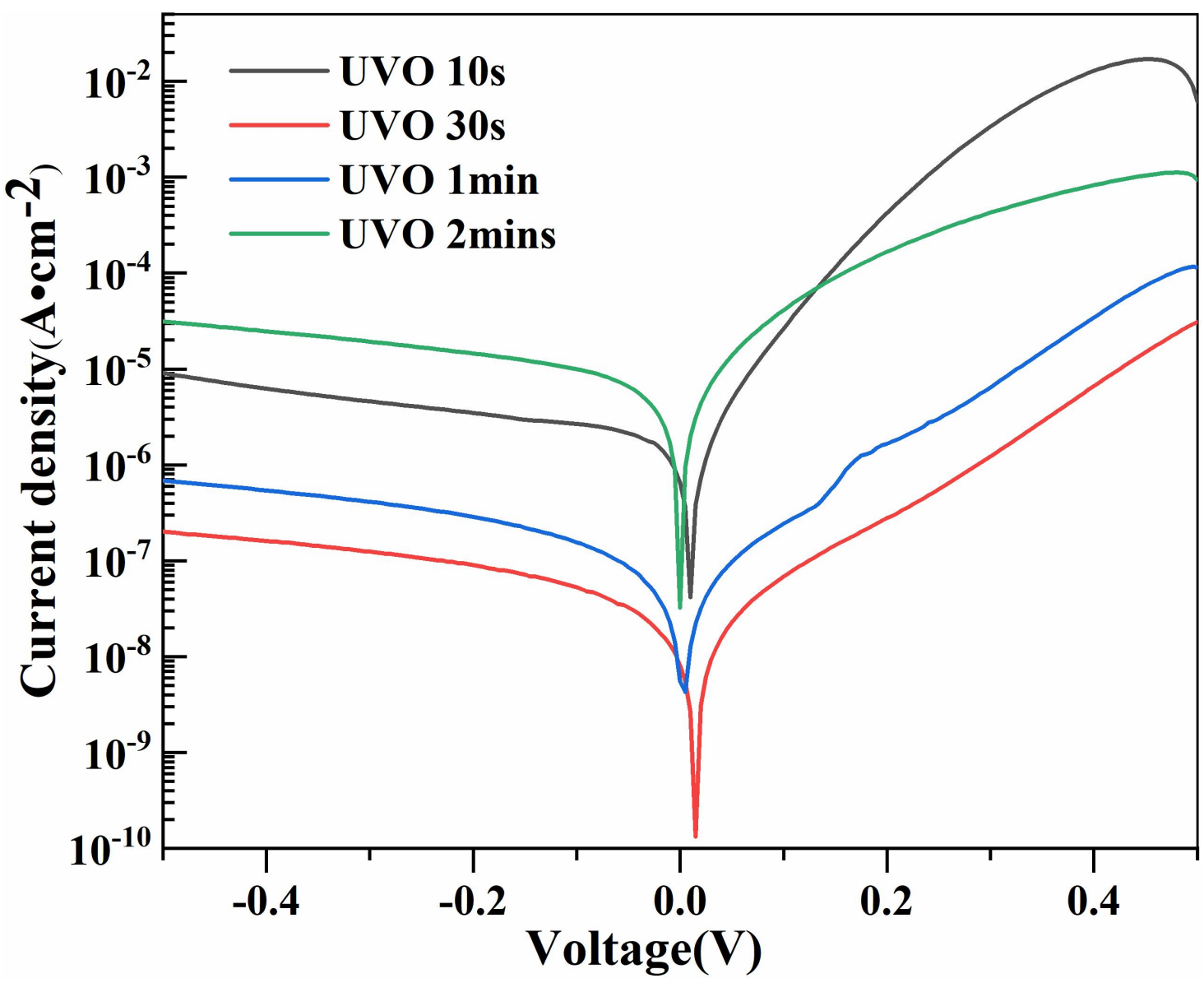

Figure S6. UVO-time-dependent J–V characteristics of Poly-TPD-buffered PbS CQD photodiodes. Devices were treated with UVO for 10 s, 30 s, 1 min, and 2 min, and compared under dark conditions. Among these conditions, the UVO-30 s treatment most effectively suppressed the dark current and was therefore selected as the optimized UVO exposure time.

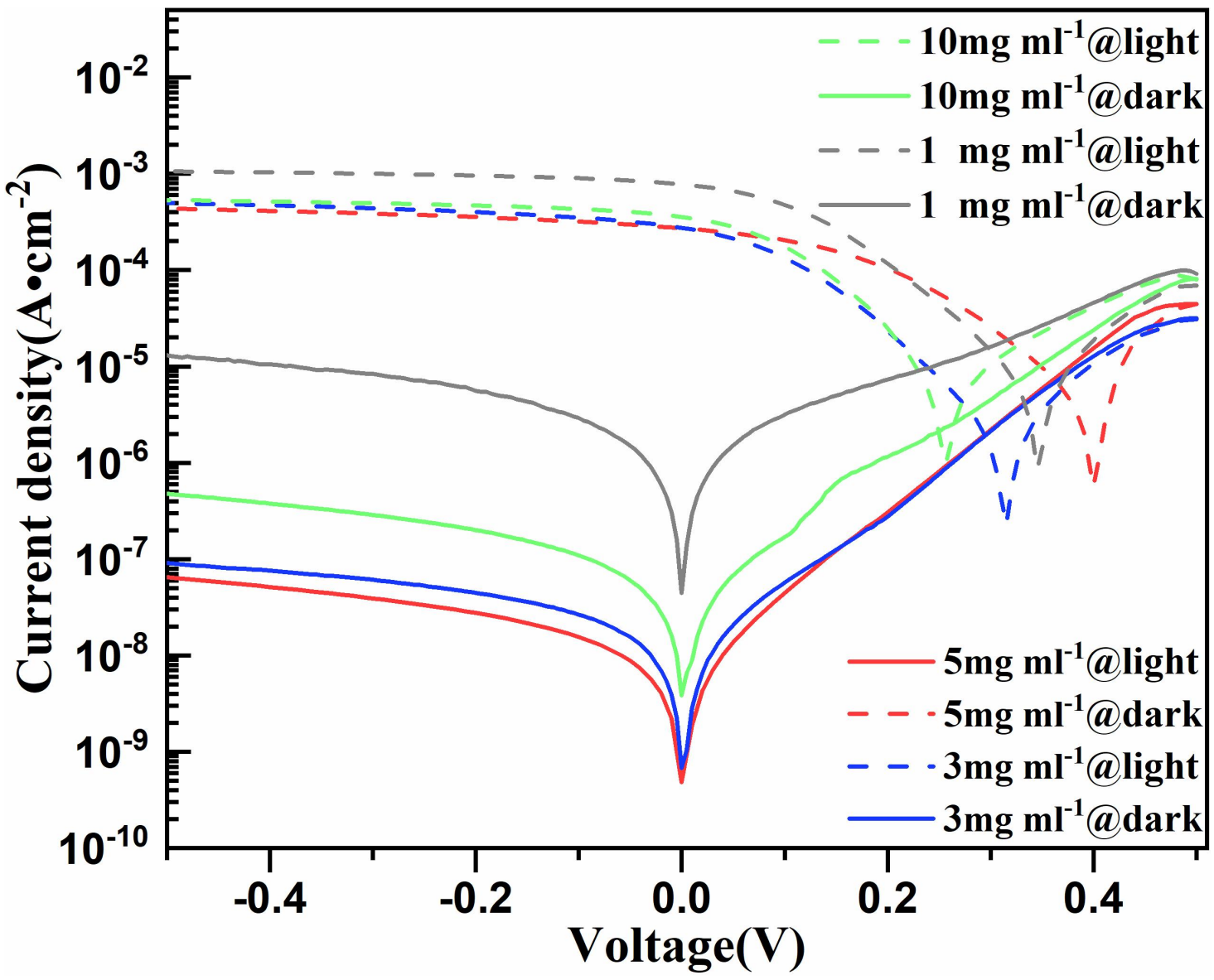

Figure S7. Thickness-dependent J–V characteristics of PbS CQD photodiodes with different Poly-TPD interlayer thicknesses. Representative dark and illuminated J–V curves of devices with Poly-TPD interlayers of approximately 3, 8, 12, and 20 nm, corresponding to Poly-TPD precursor concentrations of 1, 3, 5, and 10 mg mL^{-1}, respectively. The 5 mg mL^{-1} condition (~12 nm) gives the lowest dark current among the tested thicknesses, whereas the ultrathin 3 nm and overly thick 20 nm interlayers show inferior performance, indicating a thickness-dependent trade-off between interfacial coverage/protection and hole transport.

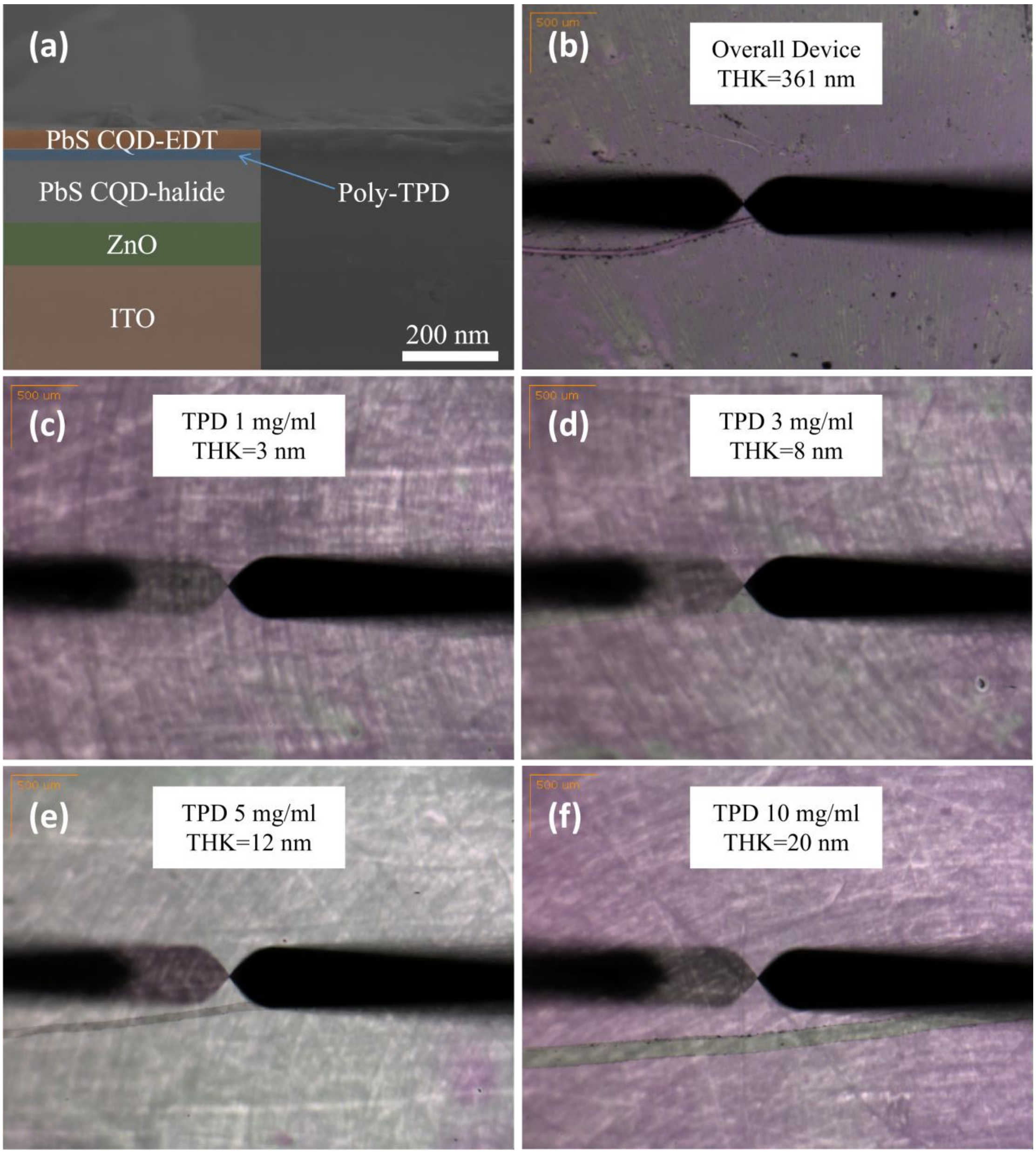

Figure S8. (a) Cross-sectional SEM image of the device. Surface profile and thickness measured by profilometry: (b) overall device (361 nm). Poly-TPD films spin-coated at different concentrations: (c) 1 mg mL^{-1} (3 nm), (d) 3 mg mL^{-1} (8 nm), (e) 5 mg mL^{-1} (12 nm), and (f) 10 mg mL^{-1} (20 nm).

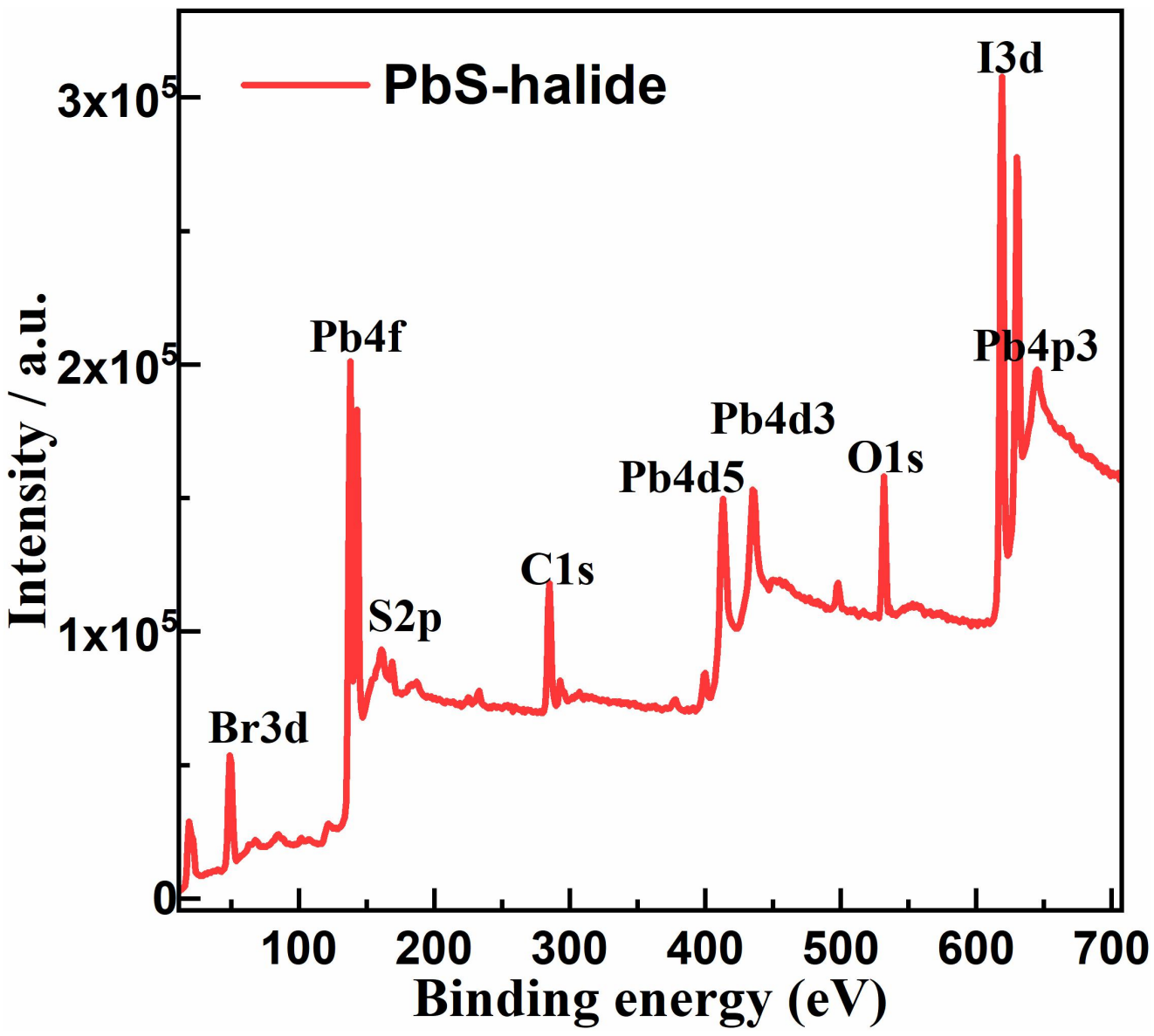

Figure S9. XPS survey spectrum of the PbS-halide film after EDT/acetonitrile treatment without the Poly-TPD buffer. The detectable Pb, S, I, and Br signals confirm that XPS can directly probe the exposed PbS-halide layer. For the Poly-TPD-covered PbS-halide sample, the XPS signal is dominated by the surface Poly-TPD layer, preventing reliable quantitative extraction of the buried PbS-halide chemical composition under conventional XPS probing depth.

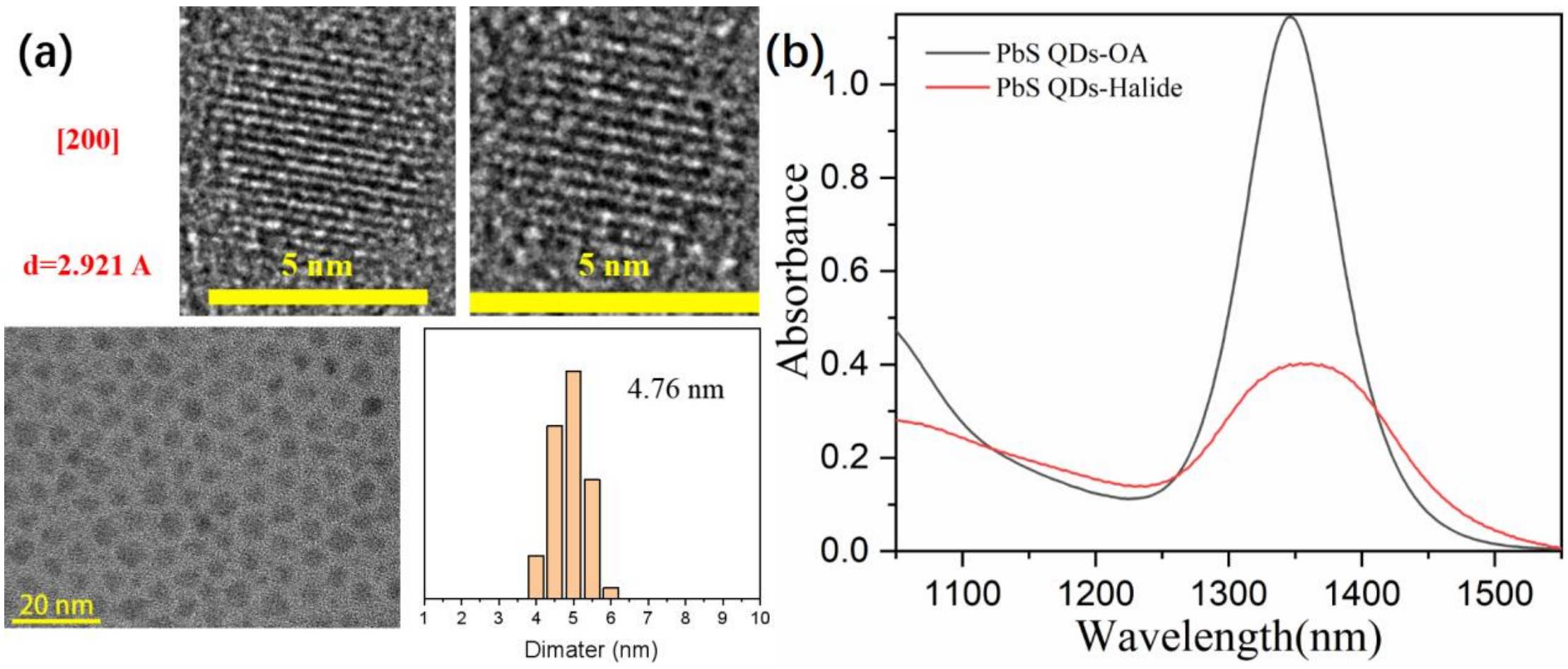

Figure S10. (a) TEM characterization of the synthesized 1350 nm PbS QDs, showing clear (200) lattice fringes with the expected interplanar spacing and an appropriate size distribution. (b) UV–vis–NIR absorption spectra of the 1350 nm PbS QDs before and after halide ligand exchange.

Table S1. Performance statistics of the previously reported PbS CQD photodiodes with absorption peak wavelength above 1350nm.

**Year/Ref.**	**λ_{abs}(nm)**	**J_{dark} (nA cm^{-2})**	**EQE (%)**	**Responsivity (A/W)**	**D^* (Jones)**
**2021**[1]	**1600**	**2480@-0.5V**	**80@-0.5V**	**0.1**	**8×10^{11}**
**2021**[2]	**1550**	**150@-0.5V ***	**70@-1V**	**0.9**	**1.6×10^{12}**
**2022**[3]	**1550**	**1200@-0.5V**	**-**	**0.77**	**5×10^{11}**
**2022**[4]	**1450**	**490@-0.5V**	**75@-0.5V**	**-**	**7×10^{11}**
**2025**[5]	**1413**	**82@-0.5V**	**33.3@-0.5V**	**0.38**	**1.2×10^{12}**
**2023**[6]	**1390**	**158@-0.5V**	**55.3@-0.5V**	**0.62**	**2.05×10^{12}**
**2022**[7]	**1450**	**1000@-0.5V***	**55.5@-0.5V**	**0.766**	**3×10^{11}**
**2023**[8]	**1550**	**100@-0.5V**	**65.6@-0.5V**	**0.82**	**6.67×10^{11}**
**2024**[9]	**1420**	**2000@-0.5V***	**56.5@-1V**	**0.65**	**1.78×10^{12}**
**This work**	**1352**	**74@-0.5V**	**38.2@-0.5V**	**0.42**	**2.1×10^{12}**

***Estimate from the original document**

References

[1] M. Vafaie, J. Z. Fan, A. Morteza Najarian, O. Ouellette, L. K. Sagar, K. Bertens, B. Sun, F. P. García De Arquer, E. H. Sargent, *Matter* **2021**, *4*, 1042.

[2] M. Biondi, M. J. Choi, Z. Wang, M. Wei, S. Lee, H. Choubisa, L. K. Sagar, B. Sun, S. W. Baek, B. Chen, P. Todorović, A. M. Najarian, R. A. Sedighian, D. H. Nam, M. Vafaie, Y. C. Li, K. Bertens, S. Hoogland, O. Voznyy, D. A. F. García, E. H. Sargent, *Adv. Mater.* **2021**, *33*, e2101056.

[3] K. Xu, L. Ke, H. Dou, R. Xu, W. Zhou, Q. Wei, X. Sun, H. Wang, H. Wu, L. Li, J. Xue, B. Chen, T. Weng, L. Zheng, Y. Yu, Z. Ning, *Acs Appl. Mater. Interfaces* **2022**, *14*, 14783.

[4] J. M. Pina, M. Vafaie, D. H. Parmar, O. Atan, P. Xia, Y. Zhang, A. M. Najarian, F. P. G. de Arquer, S. Hoogland, E. H. Sargent, *Nano Lett.* **2022**, *22*, 6802.

[5] H. Huang, Q. Yang, H. Liu, Y. Yan, X. Xiong, Y. Zhong, X. Gao, Z. Liu, W. Ma, J. Xu, S. Wang, *Small Struct.* **2025**, *6*, 2500149.

[6] Q. Yang, H. Huang, G. Xu, Y. Yuan, M. Jiang, Y. Zhong, X. Gao, J. Xu, S. Wang, *Appl. Phys. Lett.* **2023**, *123*, 213302.

[7] Y. Zhang, M. Vafaie, J. Xu, J. M. Pina, P. Xia, A. M. Najarian, O. Atan, M. Imran, K. Xie, S. Hoogland, E. H. Sargent, *Adv. Mater.* **2022**, *34*, 2206884.

[8] L. Zhang, L. Chen, J. Yang, J. Liu, S. Lu, X. Liang, X. Zhao, Y. Yang, J. Hu, L. Hu, X. Lan, J. Zhang, L. Gao, J. Tang, *Nano Lett.* **2023**, *23*, 6489.

[9] G. Choi, M. J. Lim, I. Sutcu, J. Yu, P. Malinowski, S. Y. Lee, I. Lieberman, *Appl. Phys. Lett.* **2024**, *124*, 121103.
